# Oligometastatic Disease Detection with ^68^Ga-PSMA-11 PET/CT in Hormone-Sensitive Prostate Cancer Patients (HSPC) with Biochemical Recurrence after Radical Prostatectomy: Predictive Factors and Clinical Impact

**DOI:** 10.3390/cancers13194982

**Published:** 2021-10-04

**Authors:** Carlos Artigas, Romain Diamand, Qaid Ahmed Shagera, Nicolas Plouznikoff, Fabrice Fokoue, François-Xavier Otte, Thierry Gil, Alexandre Peltier, Dirk Van Gestel, Patrick Flamen

**Affiliations:** 1 Department of Nuclear Medicine, Institut Jules Bordet, Université Libre de Bruxelles, 1000 Brussels, Belgium; qaidahmedqaid.shagera@bordet.be (Q.A.S.); fabrice.fokoue@bordet.be (F.F.); patrick.flamen@bordet.be (P.F.); 2 Department of Urology, Institut Jules Bordet, Université Libre de Bruxelles, 1000 Brussels, Belgium; romain.diamand@bordet.be (R.D.); alexandre.peltier@bordet.be (A.P.); 3 Department of Nuclear Medicine, Centre Hospitalier de l’Université de Montréal, Montréal, QC H2X 3E4, Canada; nicolas.plouznikoff@umontreal.ca; 4 Department of Radiation Oncology, Institut Jules Bordet, Université Libre de Bruxelles, 1000 Brussels, Belgium; francois.otte@bordet.be (F.-X.O.); dirk.vangestel@bordet.be (D.V.G.); 5 Department of Medical Oncology, Institut Jules Bordet, Université Libre de Bruxelles, 1000 Brussels, Belgium; thierry.gil@bordet.be

**Keywords:** PSMA, PET/CT, oligometastatic, oligorecurrence, hormone-sensitive, prostate cancer, biochemical recurrence, metastasis-directed therapy

## Abstract

**Simple Summary:**

The early treatment of oligometastatic disease (OMD) is a promising therapeutic option for prostate cancer as it has the potential of delaying androgen-deprivation therapy (ADT) and disease progression. Next-generation imaging targeting the prostate-specific membrane antigen (PSMA-PET/CT) is considered the most accurate technique for recurrent prostate cancer. Finding clinico-pathological factors predicting positivity with OMD detection on PSMA-PET/CT, as well as assessing its impact on treatment management, were the main objectives of our study. We selected a homogenous population of ADT-free prostate cancer patients with a PSMA-PET/CT performed at biochemical recurrence (BCR) after radical prostatectomy (RP). OMD was detected in 44% of patients for a total positivity rate of 60%. PSA at the moment of PET, PSAdt, and the absence of previous salvage treatment were factors predicting PSMA-PET/CT positivity with OMD. A change in clinical management occurred in more than half of the patients, mostly to perform metastasis-directed therapy after OMD detection.

**Abstract:**

Metastasis-directed therapy (MDT) in oligometastatic prostate cancer has the potential of delaying the start of androgen deprivation therapy (ADT) and disease progression. We aimed to analyze the efficacy of PSMA-PET/CT in detecting oligometastatic disease (OMD), to look for predictive factors of OMD, and to evaluate the impact of PSMA-PET/CT findings on clinical management. We retrospectively analyzed a homogeneous population of 196 hormone-sensitive prostate cancer patients (HSPC), considered potential candidates for MDT, with a PSMA-PET/CT performed at biochemical recurrence (BCR) after radical prostatectomy (RP). Multivariable logistic regression analysis was performed based on several clinico-pathological factors. Changes in clinical management before and after PSMA-PET/CT were analyzed. The OMD detection rate was 44% for a total positivity rate of 60%. PSMA-PET/CT positivity was independently related to PSA (OR (95% CI), *p*) (1.7 (1.3–2.3), *p* < 0.0001) and PSAdt (0.4 (0.2–0.8), *p* = 0.013), and OMD detection was independently related to PSA (1.6 (1.2–2.2), *p* = 0.001) and no previous salvage therapy (0.3 (0.1–0.9), *p* = 0.038). A treatment change was observed in 58% of patients, mostly to perform MDT after OMD detection (60% of changes). This study showed that PSMA-PET/CT is an excellent imaging technique to detect OMD early in HSPC patients with BCR after RP, changing therapeutic management mostly into MDT.

## 1. Introduction

Prostate cancer (PCa) is the most common cancer in men in Europe and the second worldwide with 1.4 million new cases in 2020 [1]. After initial treatment for localized disease with radical prostatectomy (RP), between 27% and 53% of patients will develop a biochemical recurrence (BCR) defined by an increase in prostate-specific antigen (PSA) level > 0.2 ng/mL [2]. As serum PSA measurement is highly sensitive, recurrence detection is performed at very early stages when the disease is rarely detectable with conventional imaging techniques (CITs), including bone scintigraphy (BS) and computed tomography (CT). CITs are not recommended at PSA levels < 20 ng/mL [3], as they present limited accuracy, with a sensitivity of only 11% [4], resulting in an enormous number of negative or inconclusive investigations.

It is, however, crucial to distinguish between localized vs. extended metastatic disease as prognosis differs completely, as well as consequent therapeutic strategies. Recurrent disease after RP can benefit from salvage radiotherapy (sRT), recommended at PSA levels < 0.5 ng/mL, where it has been demonstrated as an outcome benefit [5]. However, about 30% of patients will not respond, because the lesion responsible for the PSA increase, not previously detected, may be out of the treatment field [6]. On the other hand, multimetastatic disease will require systemic therapies based on androgen deprivation therapy (ADT) with or without novel antiandrogen drugs or docetaxel chemotherapy [7,8].

Molecular whole-body imaging with positron emission tomography/computed tomography (PET/CT) can detect active cancer sites after injection of radiotracers targeting specific cancer cells characteristics. Several radiotracers have already been used for PCa, notably ^18^F- and ^11^C-labeled choline. However, they are not recommended at PSA levels < 1.5 ng/mL, due to its limited detection rates [9].

Prostate-Specific Membrane Antigen (PSMA) is a transmembrane type II glycoprotein overexpressed in the PCa cells’ membrane in both local and metastatic lesions [10]. This over-expression is present in over 90% of PCa cells, and its function, even if not well defined, seems to be related to facilitating the growth, migration, and invasion of PCa cancer cells. New radiotracers have recently been developed using small peptides (PSMA-ligands or inhibitors) that bind to the active site of the extracellular domain of PSMA [11]. Those small molecules have excellent properties with high specificity for receptors, high permeability in solid tumors, rapid pharmacokinetics in normal tissues, and high tumor-to-background ratios, increasing the detectability even in millimetric lesions. Moreover, no host-immune response is expected as compared to PSMA antibodies. PSMA-ligands become a radiotracer when labeled with a positron-emitter isotope such as gallium-68 or fluor-18 and, after intravenous injection, whole-body PET/CT images can be obtained [12].

^68^Ga-PSMA-11 PET/CT has demonstrated higher accuracy when compared with CIT [13,14] and other PET radiotracers such as ^18^F-choline [15] and ^18^F-fluciclovine [16], even at low PSA levels where it is clinically relevant. However, most of the works published so far include heterogeneous populations of recurrent disease, including hormone-sensitive and castration-resistant patients, as well as patients with and without hormonotherapy or systemic treatments [17].

Finally, while extensive metastatic disease will need systemic therapies starting with ADT, oligometastatic disease (OMD) with up to three or five metastatic sites can be treated locally as it is considered to be an intermediate state of tumor spread with limited metastatic capacity and less aggressive behavior [18]. Treating PCa OMD with metastasis-directed therapy (MDT) has recently shown to be an effective treatment to control limited cancer spread, improving time-to-progression and avoiding or delaying the toxicity associated with the use of ADT [19,20]. Using the most accurate imaging technique to detect PCa OMD will be of utmost importance to determine which patient could benefit from MDT. Thus, the aims of our study were to evaluate the efficacy of PSMA-PET/CT in detecting OMD, to find clinico-pathological predictive factors, and to assess its clinical impact on patient management in a selected population of HSPC patients initially treated with RP and considered potential candidates for MDT.

## 2. Results

### 2.1. Patient Population

The clinical and pathological characteristics of all patients are shown in Table 1. The median (IQR) PSA at the time of PSMA-PET/CT was 1.3 (0.5–3.2) ng/mL. The medians of PSAdt and PSAvel were 8.2 (4.2–13.3) months and 0.9 (0.3–2.5) ng/mL/year, respectively. There were 114 (58%) patients with intermediate-risk PCa at diagnosis, 75 (38%) patients with high-risk PCa, and 7 patients with unknown characteristics at diagnosis.

### 2.2. Positivity Rate and Oligometastatic Disease Detection

For the 196 patients included in the analysis, PSMA-PET/CT detected at least one lesion suspicious for recurrent PCa in 117 patients and was negative in 79 patients, resulting in an overall positivity rate of 60%. The majority of cases with a positive PSMA-PET/CT presented lesions in the lymph nodes (67%), followed by prostate bed (25%) and bone (25%), with a minority of patients with visceral metastases (4%) (Table 2). A total of 86 patients (44%) presented with a maximum of three positive lesions on PSMA-PET/CT (metachronous oligorecurrence), representing 74% of all positive scans. Multimetastatic patients represented 16% of all cases (21 patients with 4–10 lesions and 10 patients with >10 lesions). The positivity rate of PSMA-PET/CT in patients with BCR before salvage treatment (including BCP and 1st BCR groups) was 60% with no significant difference (*p* = 0.887) compared to patients with BCR after sRT (59%). On the other hand, detection of OMD on PSMA-PET/CT was significantly higher in patients presenting BCR before salvage treatment compared to those with BCR after salvage treatment (86% vs. 61% of all positive PSMA-PET/CT, respectively, *p* = 0.02).

### 2.3. PSA Levels and PSA Kinetics

The 117 positive PSMA-PET/CT patients had significantly higher PSA levels (median, 0.7 vs. 2.4 ng/mL; *p* = 0.0001), higher PSAvel (median, 0.5 vs. 1.8 ng/mL/year; *p* = 0.0001), and shorter PSAdt (median, 6 vs. 9.3 months; *p* = 0.006) than the 79 negative ones. Patients with OMD on PSMA-PET/CT had significantly lower PSA levels (median, 1.5 vs. 7.8 ng/mL; *p* < 0.0001) and lower PSAvel (1.4 vs. 6.6 ng/mL/year; *p* < 0.0001) than patients with multimetastatic disease. No significant difference was found in terms of PSAdt (5.8 vs. 6.9 months; *p* = 0.45) between oligo vs. multimetastatic patients.

**Figure 1:**
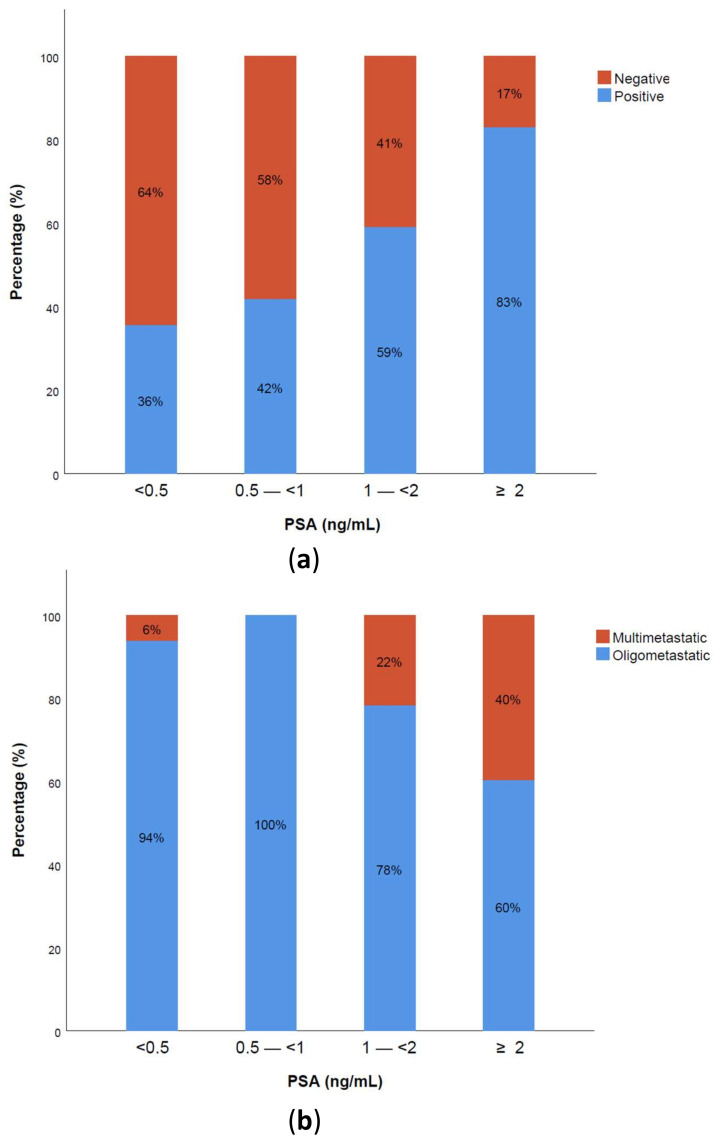
^68^Ga-PSMA PET/CT positivity rate and oligometastatic disease detection rate related to PSA levels. (**a**) Positive versus negative PSMA-PET/CT; (**b**) oligometastatic vs. multimetastatic disease detection.

PSMA-PET/CT positivity increased with higher PSA levels. The positivity distribution stratified by PSA subgroups (<0.5, 0.5–<1, 1–<2, and ≥2) is shown in Figure 1a. A substantial number of patients had a positive scan with very low PSA levels < 0.5 ng/mL (36%), while a few patients had a negative scan with high PSA levels. The higher the PSA levels, the higher the probability of finding multimetastatic disease. On the contrary, at low PSA levels < 1 ng/mL, almost all positive scans detected no more than three metastatic lesions defined as OMD, as shown in Figure 1b. 

### 2.4. Optimal Cutoff Values for PSA Kinetics

For PSA kinetics, the area under the curve (AUC) in the ROC curve analysis was 0.79 (95% CI 0.72–0.85; *p* < 0.0001) for PSAvel and 0.62 (95% CI 0.54–0.70; *p* = 0.004) for PSAdt. The optimal cut-off values for differentiating between positive and negative PSMA-PET/CT were a PSAdt of 6 months and a PSAvel of 1 ng/mL/year. There were statistically significant differences when applying the best cut-off. In 34% of patients with PSAvel < 1 ng/mL/year PSMA-PET/CT was positive while 82% of patients with PSAvel > 1 ng/mL/year had a positive scan (*p* < 0.0001). The differences in positivity rate in terms of PSAdt were also statistically significant with 74% for patients with PSAdt < 6 months and 48% for those with PSAdt > 6 months (*p* = 0.001).

### 2.5. Predictive Factors of PSMA-PET/CT Positivity and Oligometastatic Disease Detection

In the univariable analysis, PSA at the moment of PET, PSAdt, PSAvel, and T stage were factors significantly associated with an increased probability of a positive PSMA-PET/CT result, with PSA, PSAvel, and salvage treatment as factors associated with the presence of OMD (*p* < 0.05). After correlation analysis, PSAvel (ρ = 0.83) was excluded from the multivariable analysis to avoid a possible collinearity effect. 

In the multivariable analysis (Table 3), PSA and PSAdt were retained as independent predictive factors of positivity, while PSA and the absence of previous salvage treatment were independent predictive factors for the presence of OMD. Other factors included in the multivariable logistic regression analysis were not found to be significant predictors (ISUP grade group, N stage, PLND, Positive margins, and time to BCR).

### 2.6. Clinical Impact of PSMA-PET/CT in BCR

Changes in clinical management after PSMA-PET/CT are presented in Table 4. The analysis was performed for 184 patients as no data on treatment decision were available for 12 patients coming from external centers. In total, the clinical management changed after PSMA-PET/CT in 58% of patients (108/184). For all changes, 60% were related to the detection of OMD (65/108). A change in treatment was observed in 50% of BCP patients and 48% of patients with a 1st BCR. Of these two groups, initially planned sRT was modified in 33 patients by giving stereotactic ablative RT (SABR) to extraprostatic OMD, while in five patients, the treatment shifted to ADT due to multimetastatic disease detection on PSMA-PET/CT. For the group of patients with BCR after sRT, 32 of them (34%) postponed the start of ADT due to the detection of OMD that was selectively treated with MDT (SBRT in 27 patients and salvage pelvic lymph node dissection in 5 patients). In 20% of cases, a negative PSMA-PET/CT led to an active surveillance attitude, delaying the start of ADT.

## 3. Discussion

Detecting limited recurrent disease allows for tailored strategies treating the metastatic sites locally with MDT with the aim of delaying ADT and disease progression. For the success of such localized treatment, it is important to remove or encompass the whole recurrent tumoral volume, and for that, highly accurate imaging tools are needed. In this retrospective study, we analyzed the efficacy of PSMA-PET/CT in detecting recurrent OMD, looked for predictive factors, and evaluated the impact of PSMA-PET/CT findings on clinical management.

Multiple studies have evaluated the efficacy of PSMA-PET/CT to localize recurrent disease in the setting of BCR. However, most of those series presented heterogeneous populations, including castration-resistant prostate cancer patients (CRPC), patients undergoing ADT, or other systemic therapies at the time of PET imaging [21,22,23,24]. In our study, we selected a homogeneous population of HSPC patients initially treated with RP and considered potential candidates for MDT. The possible influence of ADT on PSMA expression was avoided by excluding patients that were treated with ADT. 

The overall detection rate of PSMA-PET/CT ranges from 40% to 97% mainly depending on PSA levels [25]. In our cohort, the overall positivity rate was 60%, which is in line with similar previous studies in HSPC patients [26,27,28,29]. In 44% of patients (74% of all positive scans), up to three lesions were found. When focusing on the subgroup of patients with very low PSA levels (<0.5 ng/mL), our study found a 36% detection rate. Previous studies have described detection rates ranging from 11% to 65% in that group, which can be explained by the low number of patients of some cohorts, as well as the heterogeneity of patients included. In studies including HSPC patients free from ADT, Deandreis et al. showed similar detection rates of 40% at PSA < 0.5 ng/mL [28], and Calais et al. reported a detection rate of 40% in patients scanned before sRT [30]. Two other studies on early BCR reported similar results with 34% and 36% detection rates at PSA levels < 0.5 ng/mL [26,31]. In general, studies including patients under ADT at the moment of PSMA-PET/CT showed higher detection rates [32,33]. It can be hypothesized that metastatic lesions of patients progressing under ADT will probably have more aggressive PCa clones with expected higher PSMA expression. Almost all patients (97%) with a positive PET at low PSA levels (PSA < 1 ng/mL) presented less than three lesions (OMD) with only one patient presenting multimetastatic disease in the group of patients with very low PSA < 0.5 ng/mL. It is important to remember that non-PSA-secreting metastatic prostate cancer is a rare entity with a poor prognosis, which can present with high PSMA expression levels, as previously described [34].

From the analysis of clinico-pathological predictive factors, our study found that PSA at the moment of PET and previous salvage treatment were inversely associated with the presence of OMD on a positive scan. The lower the PSA value and the absence of previous salvage treatment, the higher the probability of finding OMD. This is an important finding underlying the need of performing PSMA-PET/CT at early BCR stages where the probability of finding limited disease spread (OMD) is higher, leading to increased treatment efficacy and better outcomes. On the other hand, PSA kinetics, ISUP grade group, and other clinical variables were not associated with the presence of OMD. In our cohort, PSA and PSAdt were factors independently associated with an increased probability of a positive PET/CT. PSA level at the moment of PET is a widely accepted factor predicting PSMA-PET/CT positivity in almost all series [21], but the association with PSA kinetics is under debate. In a recent meta-analysis, there was a significant difference in PET positivity between PSAdt < 6 months compared to >6 months [35]. Interestingly, we found a PSAdt cut-off of 6 months to best differentiate between a higher (74%) and lower (48%) probability of a positive scan. 

Thirdly, treatment changes after PSMA-PET/CT were analyzed. We demonstrated an impact on clinical management in more than half of the patients (58%), agreeing with previous reports showing a clinical impact in 60% [36,37,38], and slightly higher than the 54% reported in a recent meta-analysis [39]. The most frequent reason for a treatment change in our study was OMD detection treated with MDT (60%). In 32 patients, MDT delayed the start of ADT, sparing the patient its undesirable side-effects. Moreover, delaying ADT could potentially have an important impact on national health care costs [40]. The ability of oligometastatic MDT to delay ADT and improve time to progression in PCa has been recently proven in several retrospective and prospective trials [19,20,41], and results of other ongoing prospective studies are awaited [42]. On the other hand, another prospective trial including a mixed population with different cancers presenting OMD demonstrated a benefit in OS after MDT with a median OS of 28 months in the control group vs. 41 months in the SABR group [43]. It is important to note that, at present, there is no consensus on the number of lesions to define OMD. The majority of studies have defined OMD as a maximum of either three or five metastatic lesions. In our study, we used the definition of up to three metastatic lesions, as proposed in several prospective PCa trials [19,42]. However, if multiple lesions are grouped in a single region, those could theoretically be safely treated. The consensus obtained by radiation oncologist experts using the Delphi round process regarding the maximum number of lesions that can be considered as OMD was that the maximum number must be limited by the ability to deliver safe, curative-intent MDT, which can vary on a case-by-case basis [44]. Another reason for changing therapeutic strategy in our cohort was the detection of previously unknown multimetastatic disease in five patients with BCR before sRT. This is indeed a new intermediate category of HSPC patients presenting with metastatic disease on PSMA-PET/CT but negative on CIT. Those patients have no established/validated treatment strategy yet. Systemic treatments with either taxane-based chemotherapy or novel antiandrogen drugs are approved for M_1_ HSPC with metastatic disease on CIT [7,8], while PSMA-PET/CT is now detecting metastatic disease in an earlier stage, previously considered M_0_. Future prospective studies should elucidate if multimetastatic PSMA-PET/CT HSPC patients, M_0_ on CIT, could benefit from systemic therapies. This could also include treatment with beta or alpha-emitting isotopes such as ^177^Lu-PSMA-617, which is a likely soon-to-be approved treatment for mCRPC after the positive results of the phase III Vision trial [45]. Prospective trials analyzing the combination of ^177^Lu-PSMA-617 with novel antiandrogen drugs in PSMA-positive HSPC patients are ongoing [NCT04720157].

In some cases, PSMA-PET/CT may be negative even in the presence of BCR. Possible reasons for a negative PSMA-PET/CT are the presence of slowly progressing nonaggressive disease, local relapse located adjacent to the bladder with physiologic urinary activity (which could mask small local recurrences), small disease volume below PET resolution, or the presence of undifferentiated/neuroendocrine PCa with no PSMA expression [34]. On the other hand, despite the high sensitivity (85%) and specificity (98%) of PSMA-PET/CT [14], other processes may also overexpress PSMA, probably related to the presence of PSMA expression in the endothelial cell membrane of neovessels: for example benign lesions such as Paget bone disease, vertebral hemangioma, or fibrous dysplasia [46]; and malignant lesions such as kidney cancer, breast cancer, or sarcomas [47]. 

For all treatment changes found in our study, 38% were related to a positive PSMA-PET/CT, while 20% were related to a negative one. Clinicians felt more confident putting a patient in active surveillance or giving sRT without adjuvant ADT if they had a previously negative PSMA-PET/CT. In this line, Emmett et al. demonstrated that men with negative PSMA-PET/CT results or disease still confined to the prostatic fossa at BCR after RP demonstrate a higher 3-year progression-free survival (81%), despite receiving less extensive radiotherapy and lower rates of additional ADT than patients with extrafossa disease (45%), *p* < 0.0001, and this was a better independent predictor of progression-free survival than established clinical factors [48]. Based on European Association of Urology (EAU) guidelines for BCR PCa published this year, next-generation imaging techniques are currently not recommended for patients with low-risk BCR (Gleason Score < 8 and PSAdt > 12 months), as the outcome for those patients will not differ when put into active surveillance [49]. In our cohort of 196 patients, there were 21 positive PSMA-PET/CT with clinical characteristics of EAU low-risk BCR: 10 patients with OMD treated with MDT, 7 patients with lesions confined to the prostatic bed treated with sRT, and 4 patients with multimetastatic disease treated with ADT. Of course, regulatory aspects and socioeconomic factors may also influence the decision to perform a PSMA-PET/CT as cost and radiotracer accessibility vary widely between countries.

This study was not without limitations, mostly emerging from its retrospective nature, the limited number of patients, and the lack of a comparison arm. Direct histological validation was rarely obtained. This is a known limitation in imaging studies, especially in recurrent PCa, as the biopsy of all PET-positive lesions is generally neither technically nor ethically feasible. A composite standard of reference (histopathology, clinical, and/or diagnostic imaging follow-ups) was used. In the group of patients with PSMA-PET/CT performed before sRT, possible underestimation of the local prostate bed relapse cannot be excluded, due to physiologic urinary excretion of ^68^Ga-PSMA-11. 

## 4. Materials and Methods

### 4.1. Patient Population

From a database of 400 consecutive ^68^Ga-PSMA-11 PET/CT performed at our institution for PCa recurrence detection, we analyzed a homogeneous population of 196 patients initially considered M_0_ HSPC. All included patients presented BCR as defined by the European Association of Urology [2] and were initially treated with radical prostatectomy (RP), with or without adjuvant or sRT. Patients were free from ADT and did not receive previous systemic anticancer treatment such as novel antiandrogen drugs or chemotherapy. 

PSA was measured at the time of PSMA-PET/CT, as well as PSA kinetics (doubling time, PSAdt; velocity, PSAvel). Other recorded variables (age, time since radical prostatectomy, T stage, N stage, ISUP grade group, margins after RP, adjuvant RT, and salvage treatment) are presented in Table 1. Patients were also grouped into three different clinical stages based on possible BCR scenarios: (a) biochemical persistence (BCP) defined as PSA ≥ 0.1 ng/mL at least 6 weeks after RP; (b) first-time BCR, defined as a PSA increase of >0.2 ng/mL after RP; and (c) second-time BCR with a PSA increase of >0.2 ng/mL after salvage RT.

### 4.2. Radiotracer Preparation

^68^Ga was obtained after elution from a ^68^Ge/^68^Ga radionuclide generator (Gali-Eo; IRE, Belgium) and used for radiolabeling after 5 min of incubation at room temperature by using a sterile cold kit GMP vial containing 25 µg of lyophilized precursor PSMA-11 (Telix Pharmaceuticals Ldt., Melbourne, Australia) following the manufacturer’s recommendations as previously published [50]. Quality control was performed using thin-layer chromatography showing a radiochemical purity of >99%, and sterility and pyrogen content were tested according to European Pharmacopoeia methods.

### 4.3. Imaging Procedure

Images were acquired in a single center using a General Electric (GE) Discovery 690 time of flight (TOF) PET system, 60 min after injection of 2 MBq/kg of ^68^Ga-PSMA-11 (196 ± 44 MBq). No diuretics were administered, and patients were asked to void their urinary bladder immediately prior to the scan. No fasting or special diet was required. Patients were scanned from the mid-thigh to the top of the skull in caudo-cranial orientation with raised arms. All PET scans were acquired in three-dimensional mode with an acquisition time of 2 min/bed position with an overlap of 23.4%. The images were corrected for attenuation and for scatter using the CT data. A low-dose CT (120 kV) was performed without iodine contrast injection.

### 4.4. Image Analysis

All PSMA-PET/CT images were read by two experienced nuclear medicine physicians using a dedicated workstation (Advantage Workstation; GE Healthcare, Chicago, IL, USA) with the commercial PET VCAR software AW Server 3.2, having access to clinical data and other imaging exams. Disagreements were resolved by consensus. Visual interpretation was performed following EANM standardized image interpretation recommendations considering any focal uptake of ^68^Ga-PSMA-11 higher than the surrounding background and not associated with physiological uptake, as suggestive of malignancy [51]. The number and localization of the lesions were recorded. Oligometastatic disease was defined as 3 or fewer pathologic foci, as previously proposed [19,42]. Positive lesions were validated based on a composite standard of histology, diagnostic imaging, and/or clinical follow-up. A multidisciplinary meeting composed of at least one radiation oncologist, one urologist, one medical oncologist, one radiologist, and one nuclear medicine physician decided by consensus on the subsequent treatment plan to be adopted for each patient.

### 4.5. Clinical Management Impact

Clinical impact was assessed by an experienced urologist (R.D.) blinded to PSMA-PET/CT results, ruling on the treatment that would have been applied to each patient if data from the PSMA-PET/CT were not available based on current clinical guidelines. That decision was compared to the final treatment decision taken at the multidisciplinary uro-oncology tumor board after presentation of PSMA-PET/CT results. This analysis was performed for 184 patients as no data on treatment decision were available for 12 patients coming from external centers. For BCP and 1st BCR patients (initially planned for sRT), a change was considered if the treatment decision was MDT in the case of oligometastatic disease or the start of ADT ± systemic therapy in the case of previously unknown multimetastatic disease detection. For BCR after sRT patients, initially planned for palliative ADT, treatment of oligometastatic disease with MDT was considered a change in clinical management. Finally, active surveillance was also considered a change in patients with a negative PSMA-PET/CT scan initially planned for ADT or sRT.

### 4.6. Statistical Analysis

Continuous variables are reported as medians (interquartile range, IQR) and categorical variables as relative/absolute frequencies. PSA kinetics (PSAdt and PSAvel) were calculated using the Memorial Sloan Kettering Cancer Center calculator (http://www.mskcc.org/nomograms/prostate/psa_doubling_time, accessed date on 28 August 2021). The normality of distributions was verified using a Kolmogorov–Smirnov test. The Mann–Whitney U-test was used to test for differences between positive and negative PET for continuous variables, and the chi-squared test was used for categorical variables. To better understand the distribution of PSMA-PET/CT positivity rate, patients were grouped into different intervals of PSA (<0.5, 0.5–<1, 1–<2, ≥2). Receiver operating characteristic (ROC) curves were created by plotting sensitivity vs. 1-specificity, and the best cut-off value to differentiate positive vs. negative scans was calculated using the Youden’s index. PSA kinetics (PSAdt and PSAvel) were dichotomized according to the obtained cut-offs. 

Univariable and multivariable logistic regression analysis were performed to identify independent predictive factors for scan positivity and OMD detection. Different variables were included: T stage (≥3a vs. <3a), N stage (N0 vs. N1), positive margins (yes/no), pelvic lymph node dissection (PLND) (yes/no), ISUP grade group (≥4 vs. <4), PSA levels at time of PSMA-PET/CT (ng/mL), PSAdt (≥6 vs. <6 months), PSAvel (≥1 vs. <1 ng/mL/year), time from RP to BCR (months), and previous salvage treatment (yes/no). 

A correlation analysis was performed for all continuous variables in order to identify highly correlated parameters (Spearman’s correlation ≥ 0.8) and to investigate a possible problem of multicollinearity in multivariable analysis. Only variables with *p* < 0.05 at univariable analysis and low intercorrelation were included in the multivariable analysis. The odds ratio (OR) computed by the logistic regression and its 95% confidence intervals (CI) were reported. A two-sided *p* value < 0.05 was considered as statistically significant. Statistical analyses were performed using SPSS statistics v27.0 (IBM Corp, Armonk, NY, USA).

## 5. Conclusions

This study showed the excellent capabilities of PSMA-PET/CT to detect OMD early in a selected population of HSPC patients with BCR after RP. Low PSA levels and the absence of previous salvage treatment were independent factors predicting OMD on PSMA-PET/CT. Detecting recurrent disease resulted in a change in therapeutic management in more than half of the patients, mostly by giving MDT after OMD detection. 

## Figures and Tables

**Table 1:** Clinical characteristics (*n* = 196).

Characteristics		Values
Age (year), med (IQR)		70 (64–74)
PSA at PET/CT (ng/mL), med (IQR)		1.3 (0.5–3.2)
PSAdt (mo), med (IQR)		8.2 (4.2–13.3)
PSAvel (ng/mL/year), med (IQR)		0.9 (0.3–2.5)
Time to BCR (mo), med (IQR)		52 (18–97)
pT stage, *n* (%)		
	T2a	6 (3.1)
	T2b	14 (7.1)
	T2c	59 (30.1)
	T3a	69 (35.2)
	T3b	36 (18.4)
	T4	1 (0.5)
	Unknown	11 (5.6)
pN stage, *n* (%)		
	N1	13 (6.6)
	N0	83 (42.3)
	Nx	100 (51)
ISUP grade group, *n* (%)		
	1	29 (14.8)
	2	71 (36.2)
	3	49 (25.0)
	4	24 (12.2)
	5	11 (5.6)
	Unknown	12 (6.1)
Positive surgical margins, *n* (%)		68 (34.7)
Adjuvant RT, *n* (%)		34 (17.3)
Salvage therapy, *n* (%)		75 (38.3)
Clinical Stage, *n* (%)		
	BCP	15 (7.7)
	1st BCR	80 (40.8)
	post-sRT	101 (51.5)

BCR: biochemical relapse; BCP: biochemical persistence; RP: radical prostatectomy; RT: radiotherapy, sRT: salvage radiotherapy, PSA: prostate-specific antigen, PSAdt: PSA doubling time, PSAvel: PSA velocity, ISUP: international society of urologic pathologists, n: number.

**Table 2:** Positivity rate of ^68^Ga-PSMA-11 PET/CT.

Overall Positivity Rate, Number (*n*) (%)		117 (60)
Lesion Count Per Patient, *n* (%)		
	1 lesion	57 (49)
	2–3 lesions	29 (25)
	4–10 lesions	21 (18)
	>10 lesions	10 (8)
Region-based positivity rate, *n* (%)		
	Prostatic bed	29 (25)
	Lymph Node	79 (67)
	Bone	29 (25)
	Visceral	5 (4)

**Table 3:** Univariable and multivariable logistic regression analysis.

Variables	Univariable Analysis		Multivariable Analysis	
	OR (95% CI)	*p* Value	OR (95% CI)	*p* Value
**Predictive Factors for Positive vs. Negative 68Ga-PSMA-11 PET/CT**				
Tumor stage (≥T3a vs. <T3a)	2.6 (1.4–4.8)	**0.001**	1.8 (0.8–3.7)	0.107
Nodal stage (N0 vs. N1)	1.6 (0.4–5.7)	0.440	-	-
Positive margins (yes/no)	1.5 (0.8–2.9)	0.209	-	-
PLND (yes vs. no)	0.9 (0.4–1.8)	0.890	-	-
ISUP Grade Group (<4 vs. ≥4)	1.0 (0.5–2.2)	0.862	-	-
PSA at PET/CT (ng/mL)	1.7 (1.3–2.2)	**<0.0001**	1.7 (1.3–2.3)	**<0.0001**
PSAvel (≥1 vs. <1 ng/mL/year)	8.5 (4.2–17.2)	<0.0001 *	-	-
PSAdt (≥6 vs. <6 months)	0.3 (0.1–0.6)	**0.001**	0.4 (0.2–0.8)	**0.013**
Time to BCR (months)	1.0 (0.9–1.0)	0.948	-	-
Salvage treatment (yes/no)	0.8 (0.4–1.4)	0.407	-	-
**Predictive Factors for Oligometastatic vs. Multimetastatic ^68^Ga-PSMA-11 PET/CT**				
Tumor stage (≥T3a vs. <T3a)	0.5 (0.2–1.4)	0.252	-	-
Nodal stage (N0 vs. N1)	0.3 (0.1–1.2)	0.103	-	-
Positive margins (yes/no)	0.4 (0.1–1.1)	0.105	-	-
PLND (yes vs. no)	0.8 (0.3–2.1)	0.672	-	-
ISUP Grade Group (<4 vs. ≥4)	0.6 (0.2–1.7)	0.391	-	-
PSA at PET/CT (ng/mL)	0.9 (0.8–0.9)	**0.002**	1.6 (1.2–2.2)	**0.001**
PSAvel (≥1 vs. <1 ng/mL/year)	0.06 (0.0–0.5)	0.009 *	-	-
PSAdt (≥6 vs. <6 months)	1.3 (0.5–3.2)	0.532	-	-
Time to BCR (months)	1.0 (0.9–1.0)	0.533	-	-
Salvage treatment (yes/no)	0.4 (0.1–0.9)	**0.036**	0.3 (0.1–0.9)	**0.038**

Statistically significant values (*p* < 0.05) in bold. ISUP: International Society of Urological Pathology; PSA: prostate-specific antigen; PSAdt: PSA doubling time; PSAvel: PSA velocity; PLND: pelvic lymph node dissection; BCR: biochemical recurrence; OR: odds ratio; CI: confidence interval. * PSAvel excluded from multivariable analysis to avoid a possible collinearity effect (ρ = 0.83).

**Table 4:** Changes in treatment management after PSMA-PET/CT at different clinical settings.

Clinical Stage	PSMA +/−	No Change	ADT to MDT	sRT to MDT	sRT to ADT	ADT to AS	sRT to AS
BCP	Pos (*n* = 9)	1	-	4	4	-	-
	Neg (*n* = 7)	7	-	-	-	-	-
1st BCR	Pos (*n* = 47)	17	-	29	1	-	-
	Neg (*n* = 28)	22	-	-	-	-	6
post sRT	Pos (*n* = 53)	20	32	-	-	1	-
	Neg (*n* = 40)	9	-	-	-	31	-

ADT: androgen deprivation therapy, sRT: salvage radiotherapy; MDT: metastasis-directed therapy; AS: active surveillance; BCP: biochemical persistence; BCR: biochemical recurrence, *n*: number.

## Data Availability

The data presented in this study are available on request from the corresponding author after legal agreement. The data are not publicly available, due to privacy and legal restrictions.
